# Supplementary material for: Microbial biomarker detection in shrimp larvae rearing water as putative bio-surveillance proxies in shrimp aquaculture
Source: PeerJ. 2023 May 16;11:e15201. doi: 10.7717/peerj.15201 (PMC10198154; doi:10.7717/peerj.15201)
Supplement: Supplemental Information 1 — ResI is the primary reservoir sample; ResII is the secondary reservoir sample located in the hatchery. Control is the water control without larvae. food nor antibiotic; With stands for the rearing water with erythromycin and “Without” for the rearing water without antibiotic. [file peerj-11-15201-s001.docx]

# Table SI: Alpha diversity of the water samples.

ResI is the primary reservoir sample; ResII is the secondary reservoir sample located in the hatchery. Control is the water control without larvae. food nor antibiotic; With stands for the rearing water with erythromycin and “Without” for the rearing water without antibiotic.

| **Sample** | **Condition** | **Observed** | **Chao1** | **Shannon** | **InvSimpson** | **Good’s Coverage** |
| --- | --- | --- | --- | --- | --- | --- |
| **Control_D0** | Control | 2021 | 2751.06 | 3.83 | 10.91 | 99.8 |
| **Control_D1** | Control | 1218 | 1675.74 | 3.9 | 22.71 | 99.8 |
| **Control_D2** | Control | 1256 | 1663.73 | 3.98 | 22.13 | 99.8 |
| **Control_D3** | Control | 882 | 1343.04 | 2.97 | 8.4 | 99.8 |
| **Control_D4** | Control | 1227 | 1749.38 | 3.58 | 15.28 | 99.9 |
| **Control_D5** | Control | 1153 | 1594.04 | 3.59 | 16.31 | 99.9 |
| **Control_D6** | Control | 1426 | 1914.65 | 3.3 | 12.03 | 99.9 |
| **Control_D7** | Control | 1373 | 1938.88 | 3.42 | 13.58 | 99.9 |
| **Control_D8** | Control | 1142 | 1625.88 | 3.25 | 10.31 | 99.9 |
| **Control_D9** | Control | 1340 | 1986.34 | 3.44 | 13.54 | 99.9 |
| **With_D1_A** | With antibiotic | 2463 | 3164.92 | 3.67 | 8.96 | 99.9 |
| **With_D1_B** | With antibiotic | 1754 | 2279.01 | 3.35 | 6.71 | 99.8 |
| **With_D1_C** | With antibiotic | 1979 | 2648.55 | 3.44 | 6.52 | 99.8 |
| **With_D2_A** | With antibiotic | 1290 | 1848.78 | 2.84 | 7.84 | 99.9 |
| **With_D2_B** | With antibiotic | 1209 | 1727.77 | 2.76 | 7.07 | 99.9 |
| **With_D2_C** | With antibiotic | 1175 | 1735.7 | 2.87 | 7.92 | 99.9 |
| **With_D3_A** | With antibiotic | 1499 | 1941.28 | 3.55 | 14.13 | 99.9 |
| **With_D3_B** | With antibiotic | 1185 | 1548.13 | 3.31 | 10.17 | 99.9 |
| **With_D3_C** | With antibiotic | 1339 | 1754.63 | 3.61 | 20.77 | 99.9 |
| **With_D4_A** | With antibiotic | 1339 | 1825.84 | 2.97 | 8.91 | 99.9 |
| **With_D4_B** | With antibiotic | 1186 | 1731.33 | 2.88 | 8.33 | 99.9 |
| **With_D4_C** | With antibiotic | 1326 | 1875.6 | 3.23 | 13.42 | 99.9 |
| **With_D5_A** | With antibiotic | 1148 | 1692.47 | 2.79 | 6.17 | 99.9 |
| **With_D5_B** | With antibiotic | 1109 | 1418.17 | 3.28 | 13.67 | 99.9 |
| **With_D5_C** | With antibiotic | 1158 | 1629.38 | 3.17 | 11.77 | 99.9 |
| **With_D6_A** | With antibiotic | 981 | 1399.27 | 3.67 | 12.58 | 99.9 |
| **With_D6_B** | With antibiotic | 1165 | 1642.84 | 3.11 | 12.17 | 99.9 |
| **With_D6_C** | With antibiotic | 1105 | 1735.21 | 3.36 | 13.6 | 99.9 |
| **With_D7_A** | With antibiotic | 1182 | 1700.24 | 3.27 | 11.13 | 99.9 |
| **With_D7_B** | With antibiotic | 1112 | 1436.49 | 2.67 | 4.8 | 99.9 |
| **With_D7_C** | With antibiotic | 1237 | 1669.52 | 3.43 | 13.47 | 99.9 |
| **With_D8_A** | With antibiotic | 1171 | 1804.2 | 2.56 | 6.24 | 99.9 |
| **With_D8_B** | With antibiotic | 1087 | 1681.65 | 2.66 | 7.36 | 99.9 |
| **With_D8_C** | With antibiotic | 1254 | 1801.56 | 2.84 | 8.2 | 99.9 |
| **With_D9_A** | With antibiotic | 1149 | 1650.25 | 3.08 | 7.75 | 99.9 |
| **With_D9_B** | With antibiotic | 1105 | 1682.64 | 2.93 | 8.72 | 99.9 |
| **With_D9_C** | With antibiotic | 988 | 1373.06 | 2.87 | 6.16 | 99.9 |
| **Without_D1_A** | Without antibiotic | 2089 | 2799.17 | 4.14 | 19.19 | 99.8 |
| **Without_D1_B** | Without antibiotic | 2105 | 2766.99 | 3.77 | 11.12 | 99.8 |
| **Without_D1_C** | Without antibiotic | 2090 | 2760.52 | 4.24 | 17.49 | 99.8 |
| **Without_D2_A** | Without antibiotic | 1894 | 2508.7 | 3.99 | 17.28 | 99.8 |
| **Without_D2_B** | Without antibiotic | 1862 | 2377.21 | 4.05 | 21.6 | 99.8 |
| **Without_D2_C** | Without antibiotic | 1766 | 2328.81 | 4.15 | 24.31 | 99.8 |
| **Without_D3_A** | Without antibiotic | 1606 | 2302.49 | 3.64 | 10.42 | 99.8 |
| **Without_D3_B** | Without antibiotic | 1893 | 2516.11 | 3.47 | 9.55 | 99.8 |
| **Without_D3_C** | Without antibiotic | 1848 | 2406.88 | 3.51 | 10.4 | 99.9 |
| **Without_D4_A** | Without antibiotic | 1678 | 2283.83 | 3.68 | 16.26 | 99.9 |
| **Without_D4_B** | Without antibiotic | 1514 | 2056.22 | 3.47 | 10.52 | 99.9 |
| **Without_D4_C** | Without antibiotic | 1628 | 2137.27 | 3.46 | 8.9 | 99.8 |
| **Without_D5_A** | Without antibiotic | 1591 | 2082.45 | 3.07 | 6.56 | 99.9 |
| **Without_D5_B** | Without antibiotic | 1608 | 2184.57 | 2.79 | 5.19 | 99.9 |
| **Without_D5_C** | Without antibiotic | 1408 | 1956.48 | 2.72 | 4.79 | 99.8 |
| **Without_D6_A** | Without antibiotic | 1576 | 2096.76 | 3.33 | 8.28 | 99.9 |
| **Without_D6_B** | Without antibiotic | 1522 | 2015.31 | 3.4 | 12.45 | 99.9 |
| **Without_D6_C** | Without antibiotic | 1454 | 1881.46 | 3.65 | 17.43 | 99.9 |
| **Without_D7_A** | Without antibiotic | 1786 | 2332.74 | 4.2 | 26.64 | 99.9 |
| **Without_D7_B** | Without antibiotic | 1916 | 2440.74 | 4.23 | 24.1 | 99.9 |
| **Without_D7_C** | Without antibiotic | 1790 | 2378.73 | 4.21 | 30.33 | 99.9 |
| **Without_D8_A** | Without antibiotic | 1733 | 2111.28 | 3.88 | 18.19 | 99.9 |
| **Without_D8_B** | Without antibiotic | 1245 | 1517.23 | 3.73 | 14.03 | 99.9 |
| **Without_D8_C** | Without antibiotic | 1453 | 2083.82 | 3.65 | 14.64 | 99.9 |
| **ResI** | Primary reservoir | 2430 | 2638.67 | 4.58 | 29.72 | 99.9 |
| **ResII** | Secondary reservoir | 2228 | 2857.71 | 3.33 | 6.9 | 99.9 |
