# Supplementary figures and images for: Microbial biomarker detection in shrimp larvae rearing water as putative bio-surveillance proxies in shrimp aquaculture
Source: PeerJ. 2023 May 16;11:e15201. doi: 10.7717/peerj.15201 (PMC10198154; doi:10.7717/peerj.15201)

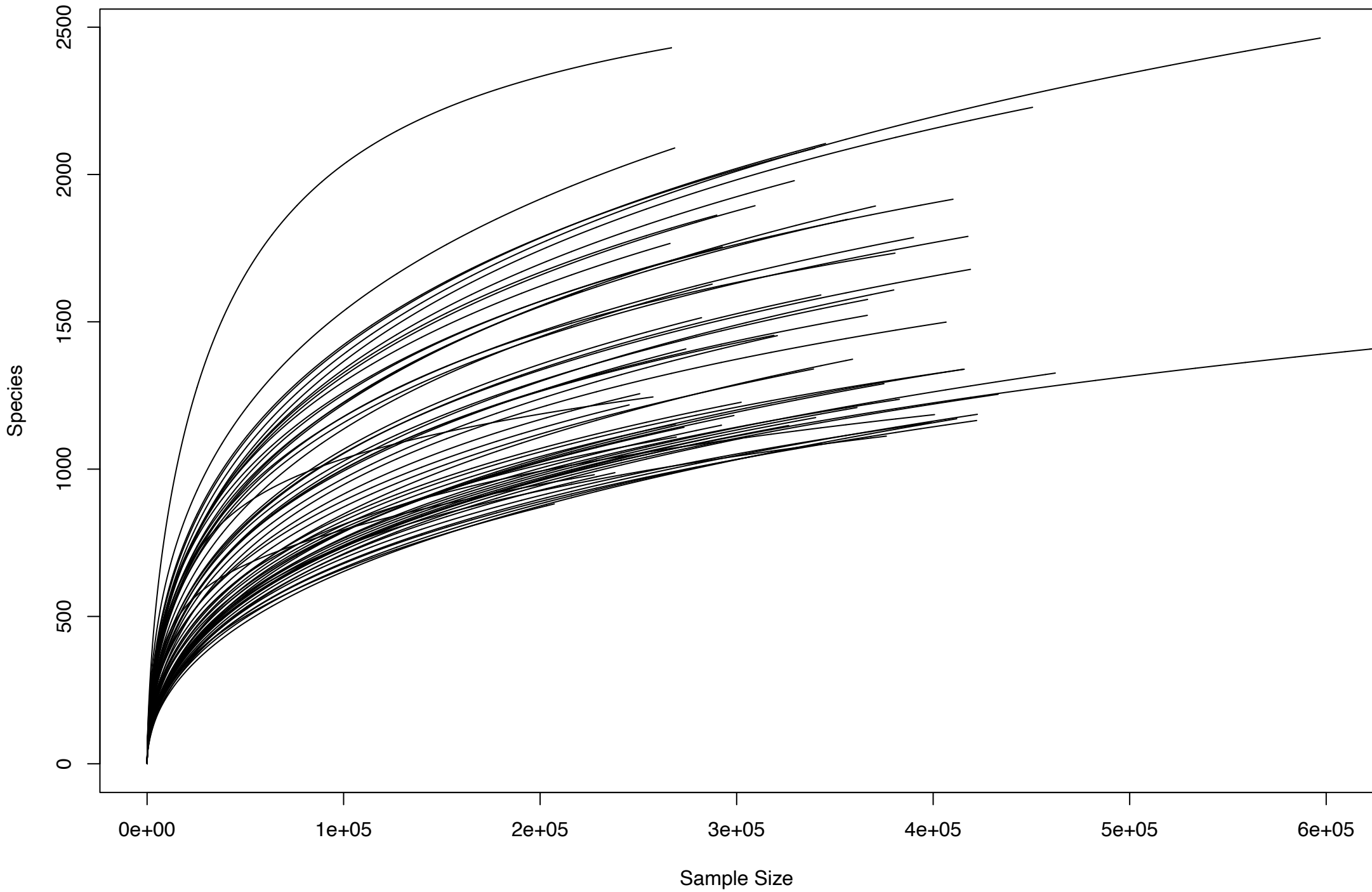

Supplement: Supplemental Information 4 [file peerj-11-15201-s004.pdf]
